# Golf course living leads to a diet shift for American alligators

**DOI:** 10.1002/ece3.10495

**Published:** 2023-08-31

**Authors:** Adam E. Rosenblatt, Robert Greco, Eli Beal, Joseph Colbert, Yank Moore, Victoria Baglin, James C. Nifong

**Affiliations:** ^1^ University of North Florida Jacksonville Florida USA; ^2^ Jekyll Island Authority Jekyll Island Georgia USA; ^3^ University of Florida Gainesville Florida USA

**Keywords:** barrier island, crocodilian, feeding pattern, Georgia, land use change, predator

## Abstract

Human‐driven land use change can fundamentally alter ecological communities, especially the diversity and abundance of large‐bodied predators. Yet, despite the important roles large‐bodied predators play in structuring communities through feeding, there have been only a few investigations of how the feeding patterns of large‐bodied predators change in human‐dominated landscapes. One group of large‐bodied predators that has been largely overlooked in the context of land use change is the crocodilians. To help fill these gaps, we studied the feeding patterns of juvenile American alligators (*Alligator mississippiensis*) on neighboring barrier islands on the southeast coast of Georgia, USA. Jekyll Island has multiple golf courses and substantial amounts of human activity, while Sapelo Island does not have any golf courses and a much smaller amount of human activity. We found that juvenile alligator populations on both islands ate the same types of prey but in vastly different quantities. Sapelo Island alligators primarily consumed crustaceans while alligators that lived on Jekyll Island's golf courses ate mostly insects/arachnids. Furthermore, the Jekyll Island alligators exhibited a much more generalist feeding pattern (individuals mostly ate the same types of prey in the same quantities) than the more specialized Sapelo Island alligators (diets were more varied across individuals). The most likely explanation for our results is that alligators living on golf courses have different habitat use patterns and have access to different prey communities relative to alligators in more natural habitats. Thus, land use change can strongly alter the feeding patterns of large‐bodied predators and, as a result, may affect their body condition, exposure to human‐made chemicals, and role within ecological communities.

## Graphical abstract

We studied the effects of golf course living on American alligator feeding patterns. We found that alligators on golf courses focus on different prey relative to alligators who do not live on golf courses, and that golf course alligators have more generalized diets at the population level.

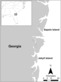

## 1 INTRODUCTION

When humans fundamentally alter a landscape to fulfill their needs and desires, the ecological communities that share the landscape are invariably altered as well. The nature of the shift depends on many factors, but the specific type of land use change that has occurred plays a major role. Some animals, like house geckos (*Hemidactylus* spp.) and common pigeons (*Columbia livia*), thrive in landscapes that have been converted into urban areas (Amoruso et al., 2014, Wessels et al., 2018), while feral swine (*Sus scrofa*) and nutria (*Myocastor coypus*) do well in landscapes altered for agriculture (Sasser et al., 2018; Seward et al., 2004). Understanding the effects of land use change on ecological communities as a whole, rather than just individual species, has never been more important. A recent analysis found that 32% of global land area has been affected by land use change just since 1960, a number that is almost four times higher than previous estimates (Winkler et al., 2021).

Golf courses are a prominent form of land use that can provide valuable habitat for many kinds of wildlife, with their relative value to wildlife increasing as anthropogenic impacts increase in surrounding areas (Colding & Folke, 2009; Hodgkison et al., 2007). Overall, the “ecological value” (i.e., animal species diversity and richness) of golf courses relative to surrounding lands is higher in 64% of cases (Colding & Folke, 2009), with birds (Merola‐Zwartjes & DeLong, 2005), turtles (Price et al., 2013), and insects (Saarikivi et al., 2015; Tanner & Gange, 2005) frequently benefitting the most. However, most studies of golf course ecology focus only on population abundance and species richness, while only a few studies have explored how predator–prey relationships can change on golf courses (e.g., Dale et al., 2020; Foley et al., 2012; Frank & Shrewsbury, 2004). Thus, a deeper understanding of ecological dynamics on golf courses is needed (Petrosillo et al., 2019).

Large‐bodied predators can be instrumental in structuring ecological communities and their behaviors can be dramatically impacted by land use change. One of the most obvious effects is in their feeding patterns. For example, diets of spotted hyenas (*Crocuta crocuta*) in peri‐urban areas of Africa and leopards (*Panthera pardus*) in agricultural areas of India are dominated by domesticated animals (Abay et al., 2011; Athreya et al., 2016). These types of diet shifts are likely caused by the increased abundance of novel prey species and the dietary adaptability of generalist predators (Bateman & Fleming, 2012). But our understanding of behavioral shifts of large‐bodied predators in landscapes that have been significantly altered by humans is still in its infancy because large‐bodied predators generally occur in low abundances or are entirely excluded throughout human‐dominated landscapes (reviewed in Bateman & Fleming, 2012).

One group of large‐bodied predators that has been mostly overlooked in the context of land use change is the crocodilians (Cavalier et al., 2021), a circumtropical group of species (Somaweera et al., 2020). Many crocodilian populations have increased during the last several decades after precipitous declines during the mid‐20th century, and some of these population rebounds have occurred in areas that are increasingly influenced by human‐driven land use change. For example, resurgent Nile crocodile (*Crocodylus niloticus*) populations in Namibia have increasingly come into conflict with humans in areas now used for cattle ranching (Aust et al., 2009), and estuarine crocodiles (*Crocodylus porosus*) in Malaysian Borneo are becoming common residents of oil palm plantations (Evans et al., 2016). While there have been a few studies of crocodilian populations in human‐dominated areas, most have aimed only to investigate population distribution and habitat use (e.g., Beal & Rosenblatt, 2020; Eversole et al., 2018; Skupien & Andrews, 2017), rates of human–crocodile conflict (e.g., Garcıa‐Grajales & Buenrostro‐Silva, 2019; Uluwaduge et al., 2018; Wallace et al., 2011), or population management techniques (Kidd‐Weaver et al., 2022). As a result, it remains unclear how crocodilian ecology, and especially feeding patterns, might shift in areas that have undergone land use change.

To help fill this knowledge gap, we investigated the potential changes in feeding patterns of crocodilians living on golf courses. We focused on American alligators (*Alligator mississippiensis*) because they are the most abundant large‐bodied predators in the southeastern United States (Mazzotti & Brandt, 1994), a region that contains almost 3800 golf courses (Lyman et al., 2007) covering approximately 270,000 ha (Moeller & Waters, 2021). Alligator population sizes have been growing for several decades and these populations often encroach onto golf courses since their natural habitats are shrinking in the face of human‐driven land use change (Johnston et al., 2010) and golf courses offer freshwater habitat in more saline coastal areas. We compared diet data from two alligator populations on neighboring barrier islands that have followed divergent land development paths. We hypothesized that alligators on the more developed island with golf courses would have diets dominated by different prey relative to alligators on the less developed island. Additionally, we expected that golf course alligators would have more homogenous diets (i.e., diets would be more similar across individuals) than alligators elsewhere. Our hypotheses were informed by the fact that available habitats on golf courses are mostly lakes and ponds, and previous research has shown alligators in lakes have access to different kinds of prey groups and have more homogenous diets than alligators in other types of habitats (Rosenblatt et al., 2015).

## 2 METHODS

### 2.1 Data collection

**FIGURE 1:**
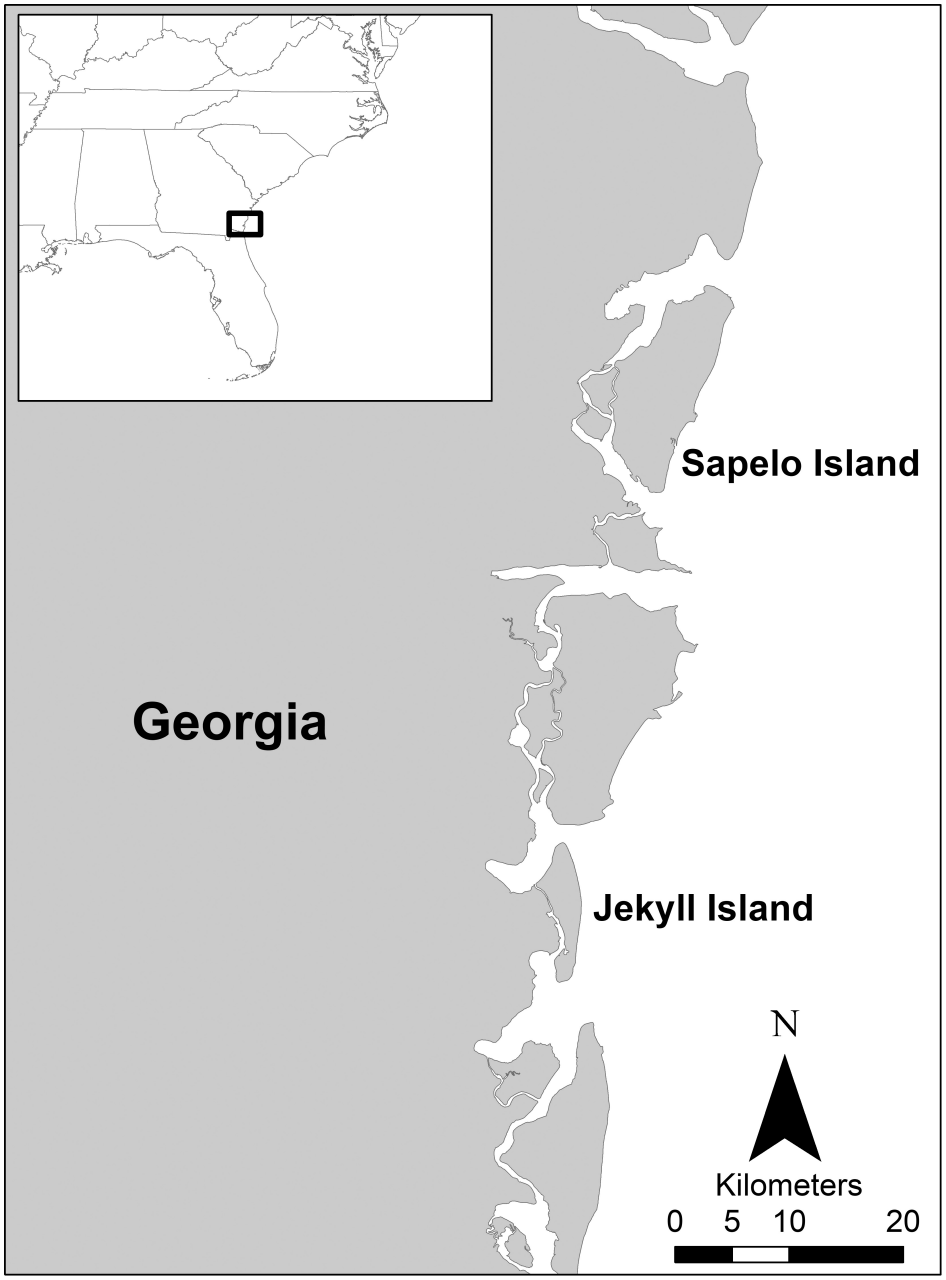
Map of Jekyll Island and Sapelo Island, the two barrier islands on the southeast coast of Georgia where alligator diet data were collected. Inset is a map of the southeast United States with a black box indicating the location of the two islands.

We conducted the study on Jekyll Island (31°3′58″ N, 81°24′52″ W), a barrier island on the southeast coast of Georgia, USA between March and August during 2019 and 2020 (Figure 1). Jekyll Island is approximately 12 km long and 3.5 km at its widest point, has four golf courses (63 holes), and is connected to the mainland via a causeway. Almost 900 people live on the island year‐round, but it also attracts over a million tourists every year. We located alligators on golf courses at night using handheld spotlights and captured them using a fishing pole and line with a modified treble hook attached. We then brought each animal to shore and taped its mouth closed before measuring total length (TL) and snout–vent length (SVL) dorsally to the nearest 0.1 cm. We determined sex via cloacal examination and then used the hose‐Heimlich technique to collect stomach contents (Fitzgerald, 1989). This technique involves filling each alligator's stomach with water and then applying pressure to the alligator's flanks until the water, and any prey items inside, exits the mouth. We collected all water and prey items in a bucket and, following release of each alligator at the capture site, sieved the prey items and stored them in jars filled with 95% ethanol. In the laboratory we identified all prey items to the lowest possible taxonomic level and weighed each item to the nearest 0.1 g (wet weight).

We compared the alligator diet data we collected on Jekyll Island with diet data collected on Sapelo Island (31°27′43″ N, 81°14′52″ W), another Georgia barrier island approximately 30 km north of Jekyll Island (Figure 1). The latter was collected between March and August during 2008 and 2009 as part of a separate study but using the same measurement and hose‐Heimlich techniques (Nifong et al., 2015). The dataset on Sapelo Island was collected 11 years prior to the dataset on Jekyll Island, but we have no reason to believe that the available prey communities on either island changed significantly during that time. As far as we know, there were no major environmental disturbances, such as hurricanes, in the region between 2008 and 2020. Sapelo Island is approximately 19 km long and 5.5 km at its widest point, has no golf courses, and is only accessible by watercraft. Around 80 people live on the island year‐round and only 20,000–25,000 tourists visit every year. Both Sapelo Island and Jekyll Island are separated from the mainland by extensive tracts of salt marsh and tidal creeks.

### 2.2 Data analysis

To determine the dominant prey categories targeted by alligators on each island, we used the Index of Relative Importance (IRI; Cortes, 1997) which is calculated as

$$\mathrm{IRI}=\%\mathrm{FO}\times \left(\%N+\%W\right),$$

where %FO is the percent frequency of occurrence of a prey category, %N is the percent of all the prey items that fall within that prey category, and %W is the percent of the wet weight of all prey items that fall within that prey category. We then converted IRI values to %IRI using the equation

$$\%{\mathrm{IRI}}_{i}=\frac{100\times {\mathrm{IRI}}_{i}}{\sum_{i}^{n}{\mathrm{IRI}}_{i}},$$

where *n* is the total number of prey categories and *i* is the focal prey category. We chose to use the IRI metric because it is not biased to the same degree as metrics based solely on %FO, %N, or %W (Cortes, 1997). Because we were unable to identify some of the recovered prey items to the family level due to advanced decomposition, we grouped prey items into 10 broad categories for analysis: fishes, reptiles, mammals, birds, crustaceans, gastropods, insects/arachnids, seeds, amphibians, and horseshoe crabs (*Limulus polyphemus*).

To assess differences in diet patterns between alligators on Jekyll Island and Sapelo Island, we used an individual specialization framework as described by Rosenblatt et al. (2015). Briefly, the framework is based on the equation TNW = BIC + WIC, where TNW is the full range of food resources used by a population, and BIC and WIC are the variance in food resource use between individuals and within individuals, respectively (Roughgarden, 1972, 1979). We divided BIC by TNW to generate an index of specialization that varied between 0 and 1, where 0 represents a population of pure generalists (individuals exactly overlap with population's resource use) and 1 represents a population of pure specialists (individuals do not overlap with other individuals in the population). Calculations of BIC/TNW are biased by individuals that only contain one prey group (Bolnick et al., 2002), so we excluded individuals who only contained one or two prey items within the same prey group and nothing else.

Specialization metrics like BIC/TNW can be difficult to compare across populations if the numbers of available prey types across different habitats are not considered. To control for this potential source of bias we converted BIC/TNW values into adjusted *E* values (*E*_adj_; Araujo et al., 2011) using the equation

$$E_{\mathrm{adj}}=\frac{E_{\mathrm{obs}}-E_{\text{null}}}{E_{\max}-E_{\text{null}}},$$

where *E*_obs_ is the observed BIC/TNW value, *E*_null_ is the average BIC/TNW value generated by a Monte Carlo resampling procedure which assumes each individual samples randomly from a shared resource pool, and *E*_max_ is the maximum possible BIC/TNW value for a given population which can be calculated as 1–1/*P*, where *P* is the number of prey categories consumed by the whole population.

We performed all analyses using R Statistical Software (v4.1.2; R Core Team, 2021) and the “rInSp” package (Zaccarelli et al., 2013). We used the package's Monte Carlo procedure to test the null hypothesis that any observed variation in diet on either island was caused by individuals sampling randomly from a shared resource pool.

## 3 RESULTS

We captured and collected stomach contents from 25 alligators living in Jekyll Island golf course ponds during 2019 and 18 during 2020. Alligators sampled ranged in size from 43.9 to 277.0 cm TL (mean = 122.6 cm ± 9.2 SE) with 36 juveniles (<180 cm TL) and seven adults (>180 cm TL). Nifong et al. (2015) captured and collected stomach contents from 54 alligators living on Sapelo Island during 2008 and 44 during 2009. These animals ranged in size from 54.6 to 315.5 cm TL (mean = 137.9 cm ± 6.1 SE) with 70 juveniles and 28 adults. Because alligators exhibit ontogenetic niche shifts between juveniles and adults (Subalusky et al., 2009), and because we were only able to sample a small number of adults on Jekyll Island, we restricted our analyses to include only juveniles on both islands. After removing recaptured individuals and those with empty stomachs, our sample sizes for Jekyll Island and Sapelo Island were 30 and 64, respectively. In the new dataset, TL of the Jekyll Island alligators (mean = 107.0 cm ± 7.0 SE) and the Sapelo Island alligators (mean = 103.5 cm ± 3.7 SE) did not differ significantly according to a Welch's *t*‐test (*p* = .67). The Jekyll Island alligators were all captured in freshwater/intermediate habitats (up to 15 ppt salinity), while 66% and 34% of the alligators on Sapelo Island were captured in freshwater/intermediate habitats and marine habitats (>15 ppt salinity), respectively.

Our %IRI analyses revealed that the two populations relied primarily on different prey categories (Table 1). More than 98% of %IRI for both populations consisted of just three prey categories (insects/arachnids, fishes, and crustaceans), but the Jekyll Island alligators relied mostly on insects/arachnids (%IRI = 0.747) while the Sapelo Island alligators relied mostly on crustaceans (%IRI = 0.514). In contrast, crustaceans were only a small portion of the Jekyll Island alligator diet (%IRI = 0.063). Furthermore, fishes made up more than double the proportion of the diet of Jekyll Island alligators (%IRI = 0.170) compared to Sapelo Island alligators (%IRI = 0.080).

**TABLE 1:** % Index of Relative Importance values of 10 prey types consumed by American alligators on Jekyll Island and Sapelo Island, Georgia.

Prey category	Jekyll Island %IRI	Sapelo Island %IRI
Insects/Arachnids	0.747	0.399
Fishes	0.170	0.080
Crustaceans	0.063	0.514
Gastropods	0.014	0.003
Seeds	0.004	0.000
Birds	0.001	0.001
Reptiles	0.001	0.0003
Mammals	0.0004	0.001
Amphibians	0.000	0.002
Horseshoe crabs	0.000	0.0005

For the BIC/TNW analyses, we had to remove more alligators from each dataset because their stomachs contained only 1–2 prey items within the same category (see methods), producing sample sizes of 27 and 54 for Jekyll Island and Sapelo Island, respectively. The BIC/TNW analyses indicated that the Sapelo Island population was much more specialized (*E*_adj_ = 0.70) than the Jekyll Island population (*E*_adj_ = 0.43), and the Monte Carlo analysis revealed that both alligator populations were not sampling randomly from a shared resource pool (*p* < .001 for both).

## 4 DISCUSSION

Converting land into golf courses can affect animal species richness and population abundance, but knowledge of how large‐bodied predator feeding habits are affected is generally lacking. We investigated how the diets of juvenile alligators are impacted by living on golf courses through a comparison of stomach contents from alligator populations living on neighboring barrier islands in southeast Georgia, USA. The most important prey group for juvenile alligators on the island without golf courses (Sapelo Island) was crustaceans, followed by insects/arachnids and distantly by fishes, while juvenile alligators on golf courses (Jekyll Island) focused heavily on insects/arachnids, followed distantly by fishes and crustaceans. Furthermore, the Jekyll Island alligators exhibited a much more generalist feeding pattern than the Sapelo Island alligators.

Our results can be explained primarily by two factors related to land use change: shifts in alligator habitat use and shifts in prey abundance. First, alligators are known to alter their habitat use in response to the presence of golf courses. On Sapelo Island, where there are no golf courses, adult male alligators tracked with GPS transmitters spent 54.5% of their time in marine habitats and 45.5% of their time in freshwater habitats (Nifong & Silliman, 2017). In contrast, on Jekyll Island adult male alligators tracked with GPS transmitters spent 58% of their time in golf course ponds and only 25.7% of their time in marine habitats, with the remaining 16.3% of their time spread across freshwater wetlands, culverts, and forest (Skupien et al., 2016). The divergent habitat use patterns on the two islands could indicate that alligators have a preference for large ponds like those found on golf courses, or alligators avoid crossing highly fragmented human‐dominated landscapes with little cover. Though the alligator habitat use patterns on Sapelo Island and Jekyll Island have only been described for adults and our study focused on juveniles, previous research has shown that large juveniles (>79 cm TL; Nifong et al., 2015), who made up more than 70% of both our datasets, can roam just as widely as adults (McNease & Joanen, 1974).

Second, if alligators on golf courses spend more time in ponds than their counterparts in natural settings, then they are likely selecting from different prey communities. Crustaceans are a highly diverse and abundant group of animals on Georgia's barrier islands (Dorjes, 1977; Prezant et al., 2002), especially in the salt marshes and brackish wetlands normally frequented by alligators, but golf courses have the potential to impact local crustacean communities. The topography and hydrology of the landscape are both affected by golf course construction (Winter & Dillon, 2005), which may alter habitats on which crustaceans rely, and pesticides used on golf courses can be acutely toxic to crustaceans (DeLorenzo et al., 2014; Rainwater, 2002). Conversely, multiple studies have reported that golf courses can support higher insect species richness and abundance than nearby habitats (Tanner & Gange, 2005, Mata et al., 2017, Saarikivi et al., 2015). These trends, coupled with the altered habitat use patterns of alligators on golf courses, are likely the biggest factors causing the shift in diet we observed where Jekyll Island alligators rely most heavily on insects/arachnids, while Sapelo Island alligators focus more on crustaceans. The abundance of prey on golf courses may in fact be the main reason for the altered habitat use patterns of Jekyll Island alligators in the first place.

We also observed that Jekyll Island alligators exhibited more generalist feeding habits than Sapelo Island alligators. This means that individual alligators residing in golf course ponds were eating mostly the same types and quantities of prey, while alligators on Sapelo Island had more distinct diets from one individual to another. This is consistent with previous research on alligator feeding habits which indicated that alligator populations in lakes have more generalist feeding patterns than alligator populations in coastal habitats (Rosenblatt et al., 2015). The hypothesized causes of this phenomenon are the same ones that we identified in this study: less use of habitats outside the lakes/ponds and lower prey diversity within the lakes/ponds (Rosenblatt et al., 2015). Our results are also broadly in line with research on reptile feeding habits in other types of human‐dominated landscapes. In a meta‐analysis of the effects of urbanization on the diets of vertebrate predators, researchers found that reptiles exhibited the highest sensitivity to urbanization as dietary species richness decreased more for reptiles than any other predator group (Gámez et al., 2022).

Our results raise important questions about how alligator health and development may be impacted by living on golf courses. Are juvenile alligator body condition and growth rates affected by consuming more insects/arachnids on golf courses than crustaceans? Do alligators on golf courses bioaccumulate pesticides that target insects/arachnids? The first question could be answered by weighing captured alligators while also measuring length to calculate body condition (Kruis et al., 2023), and by recapturing alligators repeatedly over a number of years to determine growth rates. The second question could be answered by collecting blood and muscle tissues from captured alligators for pesticide analysis. Alligators have been shown to bioaccumulate heavy metals and pesticides in other contexts (e.g., Jagoe et al., 1998; Milnes & Guillette Jr., 2008), so it would not be surprising if similar scenarios were occurring on golf courses. In fact, one study that examined chemical contaminant loads in alligators across South Carolina and Florida found that alligators captured on or near golf courses on Kiawah Island, South Carolina had the second highest contaminant loads of all the sampled populations (Bangma et al., 2017).

Alligators are very adaptable predators with flexible diets (Rosenblatt & Nifong, 2018), so it is no surprise that they can shift their feeding habits and tolerate living on golf courses, and potentially thrive in these settings. However, the long‐term health of alligator populations, and large‐bodied predator populations more broadly, in human‐dominated landscapes remains an open question. We must move beyond simply documenting their presence/absence across different environmental contexts and seek a deeper understanding of the behavioral, physiological, and evolutionary consequences of living in environments so thoroughly controlled by another species. Alligators and other crocodilians represent ideal model species for these kinds of studies because they still exist in relatively large numbers in some parts of the world dominated by humans. Our hope is that through more study and informed landscape management, humans can learn to live alongside these large‐bodied predators for the benefit of both species.

## AUTHOR CONTRIBUTIONS

**Adam E. Rosenblatt:** Conceptualization (lead); data curation (equal); formal analysis (supporting); funding acquisition (equal); investigation (equal); methodology (lead); project administration (lead); supervision (lead); validation (lead); visualization (lead); writing – original draft (lead); writing – review and editing (equal). **Robert Greco:** Data curation (equal); formal analysis (lead); investigation (equal); writing – review and editing (equal). **Eli Beal:** Data curation (equal); investigation (equal); writing – review and editing (equal). **Joseph Colbert:** Investigation (equal); writing – review and editing (equal). **Yank Moore:** Investigation (equal); writing – review and editing (equal). **Victoria Baglin:** Investigation (equal); writing – review and editing (equal). **James C. Nifong:** Investigation (equal); writing – review and editing (equal).

## CONFLICT OF INTEREST STATEMENT

The authors declare no competing interests.

## Data Availability

The Jekyll Island diet dataset is available via Dryad at doi: 10.5061/dryad.sxksn0387.
